# Fish mtDNA COI sequences from local fish markets in the Red Sea and Arabian Sea: A reference dataset

**DOI:** 10.3897/zookeys.1291.181633

**Published:** 2026-09-04

**Authors:** Nayeli G. Jiménez Cano, Agnès Dettaï, Hanan Mansoor Al-Nabhani, Mohamed Abu El-Regal, Azzah Al-Jabri, Philippe Béarez

**Affiliations:** 1 Muséum National d’Histoire Naturelle, UMR 7209 BioArch CNRS-MNHN, 75005 Paris, France Muséum National d’Histoire Naturelle, UMR 7209 BioArch CNRS-MNHN Paris France; 2 Department of Anthropology, University of California San Diego, 92093 La Jolla, CA, USA Department of Anthropology, University of California San Diego La Jolla United States of America; 3 Muséum National d’Histoire Naturelle, UMR 7205 ISYEB MNHN-CNRS-SU-EPHE-UA, 75005 Paris, France Muséum National d’Histoire Naturelle, UMR 7205 ISYEB MNHN-CNRS-SU-EPHE-UA Paris France; 4 Natural History Museum of Oman, Al-Khuwair, Muscat, Oman Natural History Museum of Oman Muscat Oman; 5 Marine Science Department, Faculty of Marine Science, King Abdul-Aziz University, Jeddah, Saudi Arabia Marine Science Department, Faculty of Marine Science, King Abdul-Aziz University Jeddah Saudi Arabia

**Keywords:** Fish diversity, mitochondrial DNA, molecular identification, Oman, Saudi Arabia

## Abstract

This data paper describes a dataset of mitochondrial cytochrome c oxidase subunit I (COI) sequences from 51 fish specimens obtained from local fish markets in eight coastal localities in Oman and Saudi Arabia. For each vouchered specimen, basic metadata such as locality, date of purchase, and morphometric data was documented, and a photographic record was made. Tissue samples were collected for DNA analysis. The COI gene was amplified and sequenced using standard DNA protocols. The resulting COI sequences were curated and deposited in the Barcode of Life Data System (BOLD). The dataset enhances the genetic representation of marine fishes from the Red Sea and Arabian Sea and adds three novel fish COI records in BOLD, *Plectropomus marisrubri*, *Scarus zufar*, and *Scorpaenopsis lactomaculata*, as well as numerous fish taxa which are currently poorly documented. These sequences represent a reference resource for specimen identification and verification, and can be used to underpin taxonomic revisions, comparative phylogenetic and phylogeographic studies, assessments of regional biodiversity and sea food authentication. In making these data freely available, this contribution promotes reproducibility, data reuse, and collaboration among researchers working on marine ecology, conservation, and fisheries management, as well as monitoring and consumer protection.

## Introduction

Fishes around the Arabian Peninsula have considerable economic importance due to their role in local and commercial fisheries (Palomares et al. 2021). However, unlike well-studied regions with extensive genetic repositories, genetic data available for fish species in this region is uneven. Most studies of fish genetics of this region have focused on the Persian Gulf (Rabaoui et al. 2019; Ludt et al. 2020) and the Red Sea (Trivedi et al. 2014; Hassan et al. 2020; Santanumurti et al. 2024), whereas efforts in Oman are limited and concentrated on a few commercially important fish families (Al-Amry et al. 2024; Elaswad et al. 2026). Given the incomplete genomic data available from this region, this gap is concerning, especially as fish stocks in the region are reported to be declining due to overfishing, habitat degradation, and climate change. In this sense, contributing to Findable Accessible Interoperable and Reusable (FAIR) genomic sequences from a relatively undersampled region should allow researchers to integrate these data into broader comparative studies at regional scale, which may be useful for revealing genetic relationships, migration patterns, and potential evolutionary adaptations of marine fish species across the Indian Ocean and Arabian Sea, contributing valuable insights into global marine biodiversity.

In this paper, we present a dataset of 51 cytochrome c oxidase subunit I (COI) sequences to support accurate specimen identification and enhance genetic representation of marine biodiversity in the Arabian Peninsula. Included are the addition of three previously unsequenced species, *Plectropomus marisrubri*, *Scarus zufar*, and *Scorpaenopsis lactomaculata*, representing novel records in the Barcode of Life Data System (BOLD). Our study lays the groundwork for future taxonomic, ecological, conservation, and biogeographical research in the region. The sequences have been deposited in BOLD, which provides a standardized, accessible database that will allow other scientists to download, analyse, and integrate these sequences into their studies, thereby supporting reproducibility and collaboration within the scientific community.

## Project description

This dataset forms part of the genetic component of PRISTINE (Prehistoric Fisheries Assisting Marine Baselines), a Marie Skłodowska-Curie Actions (MSCA) postdoctoral project integrating the study of archaeological fish remains (ichthyoarchaeology) with the study of ancient proteins (paleoproteomics) to investigate long-term patterns of fish biodiversity and exploitation in the Indo-Pacific region. Because ancient DNA preservation is often poor in tropical contexts, the project relies on collagen-based taxonomic identification as an alternative molecular approach. A key initial objective was therefore to build a genetically validated Reference Collagen Database (RCD) against which collagen spectra from archaeological fish remains can be reliably compared. The project’s sampling workflow includes the collection of fresh fish, preparation of skeletons to allow ichthyoarchaeological analyses, collection of scales to constitute the RCD, and sampling of fin tissue for molecular identification of reference specimens using COI gene sequences. The fish COI sequences generated for the RCD are presented in this data paper. This contribution expands region-specific genetic coverage, adds first sequence data for three fishes from the Arabian Peninsula, and provides a valuable resource for PRISTINE-related research as well as broader studies in fisheries science, taxonomy, and marine biodiversity.

## General description

**Figure 1.**
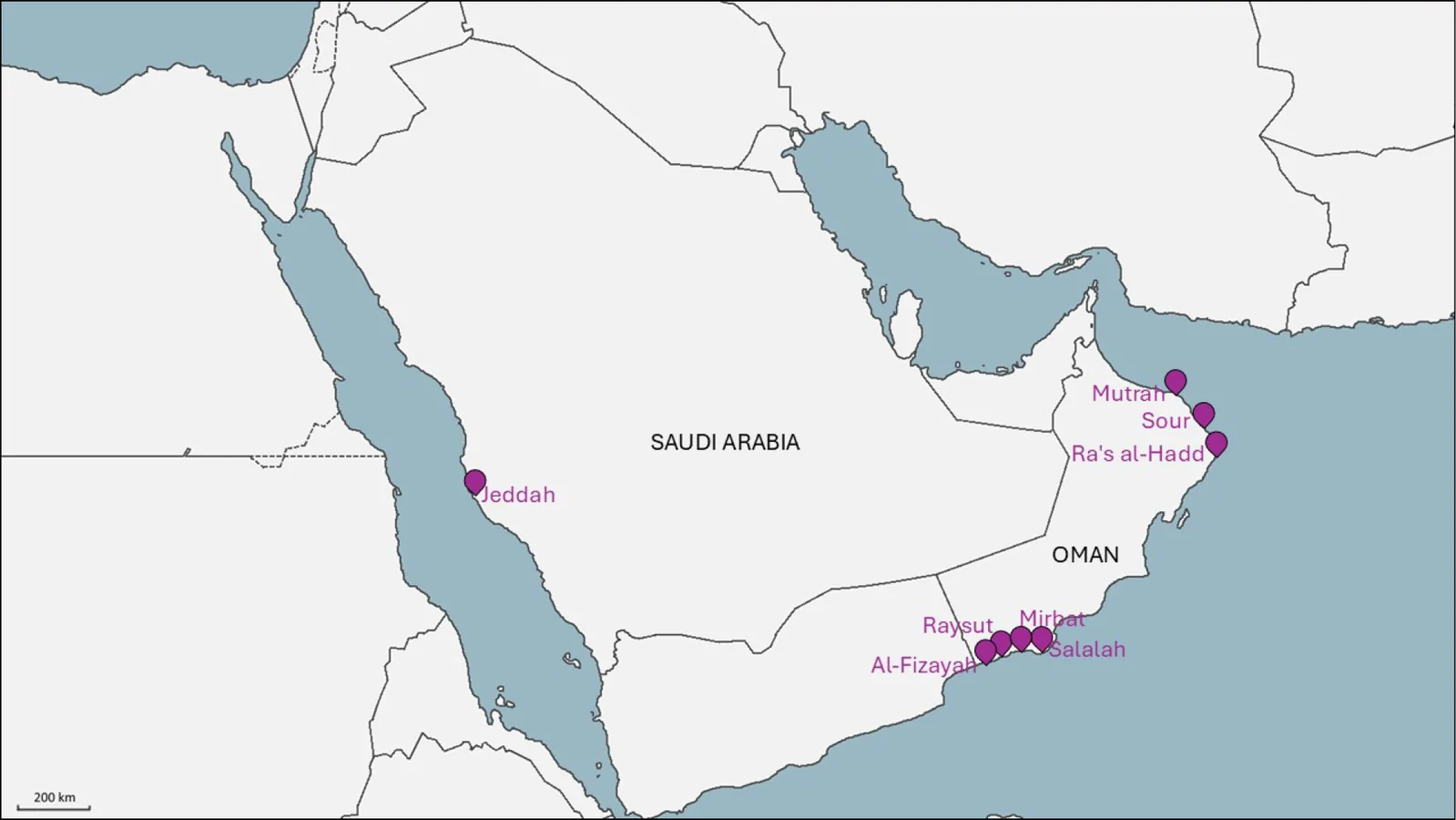
Map showing the locations sampled for this dataset.

Fish specimens were obtained from fish markets at eight coastal localities on the Arabian Peninsula, both in Saudi Arabia and Oman (Fig. 1). Particular attention was paid to the origin of specimens collected at major markets such as those in Jeddah and Muscat, either by approaching fishermen directly as they landed their catch, or by selecting very fresh fish whose origin had been carefully verified with the sellers. This ensured that the fish came from small-scale fisheries located near these markets. The material comprises 40 specimens representing 37 species from Oman and 11 specimens representing 11 species from Saudi Arabia.

Each specimen was photographed, measured, weighed, and fin-clipped. Fin clips were immediately preserved in 96% ethanol to prevent DNA degradation and stored at −20 °C until DNA extraction.

Fish specimens were preliminary identified with great care to ensure taxonomic accuracy. This process involved detailed morphological examination, including meristic and morphometric characteristics, colouration patterns, and other diagnostic features. Specialized scientific literature was consulted throughout the identification process to ensure consistency and reliability (Randall 1983, 1995; Heemstra et al. 2022). When necessary, comparisons were also made with verified museum specimens and authoritative taxonomic descriptions (Randall and Hoese 1986; Moore et al. 2011; Miyamoto et al. 2020). This rigorous approach provided a solid foundation for subsequent genetic analyses and strengthened the reliability of species-level identifications.

We used the cetyl trimethyl ammonium bromide (CTAB) method of DNA extraction, a widely employed protocol that provides high-quality DNA suitable for PCR amplification and sequencing. The protocol includes samples incubation with 600 μl of CTAB buffer and 1 μl of Proteinase K at 60 C overnight to ensure complete lysis and breakdown of cellular proteins. The aqueous phase was carefully collected after centrifugation and 600 μl of chloroform-isoamyl alcohol (CIA) 96:4 was added to separate DNA from other cellular components. Tubes were agitated 2 or 3 times to create an emulsion, and centrifugated for 2 min at 8000 rpm. Supernatant was collected and DNA was precipitated using 400 μl of isopropanol at −20 °C during at least 6 h. Sample tubes were centrifuged, and isopropanol was discarded. Pellet washing was performed by adding 500 μl of cold 76% ETOH and centrifuged for 10 min at 13500 × g. The supernatant was discarded, and the pellets were air-dried and resuspended in 50 μl TE buffer (Tris-EDTA).

The data set is composed of COI sequences that were obtained through Sanger and NGS Illumina sequencing. In the latter case, only the COI gene was included in the dataset because complete mitochondrial genomes were not recovered.

For barcoded specimens, COI amplifications were carried out in a 20 μL reaction mixture containing 15.44 μl of H_2_O, 2 μl of tampon TAQ, 1 μl of DMSO, 1 μl of BSA, 0.8 μl of dNTP, 0.32 μl of each primer, 0.8 μl of TAQ and 1 μl of the extracted DNA. For mitogenomes the mixture reaction of 20 μl consisted of 10.91 μl of H2O, 4.2 μl of LongAmp Taq Buffer 5×, 1.2 μl of dNTPs (6.6 mM), 0.97 μl of BSA (5 mg/mL), 0.6 μl of DMSO, 0.42 μl of each primer (10 pM μl), 0.36 μl of NEB LongAmp Taq and 1 μl of extracted DNA. Primers used for each specimen are shown in Table 1.

**Table 1.** List of primers used in this dataset.

**BOLD ID**	**Forward Primer**				**Reverse Primer**	
	**Name**	**5'-3' sequence**	**Source**	**Name**	**5'-3' sequence**	**Source**
PRIS007-24	16SF2009	GCCTGTTTAYCAAAAACAT	Kocher et al. 1989	MtH7061	GGGTTATGTGGCTGGCTTGAAAC	Hinsinger et al. 2015
PRIS009-24						
PRIS011-24						
PRIS013-24						
PRIS014-24						
PRIS020-24						
PRIS022-24						
PRIS024-24						
PRIS025-24						
PRIS052-24						
PRIS053-24						
PRIS016-24						
PRIS018-24						
PRIS017-24				R7146raj	AGTGGTNATGTGGTTGGCTTGAAAC	This study
PRIS021-24	Fish F1	TCAACCAACCACAAAGACATTGGCAC	Ward et al. 2005	Fish R1	TCAACCAACCACAAAGACATTGGCAC	Ward et al. 2005
PRIS027-24				FishR2	ACTTCAGGGTGACCGAAGAATCAGAA	
PRIS038-24						
PRIS043-24						
PRIS050-24						
PRIS051-24						
PRIS005-24	Fish F2	TCGACTAATCATAAAGATATCGGCAC				
PRIS006-24						
PRIS028-24						
PRIS032-24						
PRIS003-24	Fish-BCL	TCAACYAATCAYAAAGATATYGGCAC	Baldwin et al. 2009	Fish-BCH	TAAACTTCAGGGTGACCAAAAAATCA	Baldwin et al. 2009
PRIS004-24						
PRIS042-24	Tel F1	TCGACTAATCAYAAAGAYATYGGCAC	Dettai et al. 2011	FishR2	ACTTCAGGGTGACCGAAGAATCAGAA	Ward et al. 2005
PRIS001-24				Tel R1	ACTTCTGGGTGNCCAAARAATCARAA	Dettai et al. 2011
PRIS002-24						
PRIS008-24						
PRIS010-24						
PRIS012-24						
PRIS015-24						
PRIS019-24						
PRIS023-24						
PRIS026-24						
PRIS029-24						
PRIS030-24						
PRIS031-24						
PRIS033-24						
PRIS034-24						
PRIS035-24						
PRIS036-24						
PRIS037-24						
PRIS040-24						
PRIS041-24						
PRIS044-24						
PRIS045-24						
PRIS046-24						
PRIS047-24						
PRIS048-24						
PRIS049-24						

PCR thermal cycling profile was performed in a GeneAmp System 2700 (Applied Biosystems) thermal cycler; for barcoding specimens, the PCR protocol consisted of an initial denaturation for 1 min at 94 C, followed by 35 cycles consisting of 94 C for 30 s, 50 C for 30 s, and 72 C for 1 min, followed by a terminal elongation of 2 min at 72°, while for mitogenomes long PCRs included initial denaturation of 30 s at 94 C, followed by DNA was amplification through 60 cycles of 20 s at 94 C, 30 s at 58 C, and 14 min at 65 C, with a terminal elongation for 13 min at 65 C.

All PCR products were checked by electrophoresis on 2% agarose gels for short PCRs and in 0.8% agarose gel for long PCR products. Sequencing was performed on an automated Sanger sequencer and Next Generation Sequencing (NGS) using an Illumina sequencer producing high-quality reads using a double-multiplexing approach in which sequences of several distant teleosts, elasmobranchs, foraminifers, crustaceans, insects, ferns, and mammals were included in the library.

Raw Sanger sequences were imported into Geneious Prime 2024.0.5 and screened for quality. Low-quality reads or ambiguous bases were manually inspected. Forward and reverse sequences were aligned, and overlapping regions were trimmed to create a consensus sequence for each specimen. Reads were mapped on references (Table 2) from GenBank with Geneious Prime, and the consensus sequences were quality controlled and annotated using MitoAnnotator (Iwasaki et al. 2013).

**Table 2.** BOLD specimen IDs and corresponding reference sequences used for read mapping.

**BOLD ID**	**Mapping reference**
PRIS007-24	NC_033497
PRIS009-24	NC_028406
PRIS011-24	NC_028406
PRIS013-24	NC_011936
PRIS014-24	NC_011936
PRIS020-24	KU191261
PRIS022-24	NC_024026
PRIS024-24	NC_057057
PRIS025-24	OQ476208
PRIS052-24	NC_072284
PRIS053-24	NC_011599

The consensus sequences were compared against the Barcode of Life Database (BOLD) and NCBI’s GenBank using BLAST to check specimen identification based on sequence similarity and to confirm and refine preliminary species-level identification. Where morphological and molecular identifications were inconsistent or uncertain, the specimens were morphologically re-evaluated and the taxonomic assignments were reassessed considering published species distributions, regional occurrence records, and the provenance of the samples. Only identifications that were considered robust following this integrative review were retained in the final dataset. Cleaned COI sequences, along with their metadata (i.e. species ID and sampling location), were exported in FASTA format for submission to BOLD Systems.

## Data description

Table 3 summarizes the specimens that composed the dataset, which includes accession numbers in BOLD Systems, Museum ID numbers, specimen identification, COI length, sequencing technology, and geographic origin, including country and locality. The BOLD accession numbers offer unique identifiers within the BOLD Systems database. In BOLD, this identifier allows to freely consult supplementary information of each specimen such as photographs, primers used, and electropherograms to provide quality control for identifications and sequences. The Museum ID numbers correspond to the catalogue numbers of the Muséum National d’Histoire Naturelle (MNHN), where voucher skeletal reference specimens are stored, except for the two *Sphyrna lewini*, which were not collected due to their large size. Identification refers to the taxonomic identifications based on morphological and genetic data, allowing for accurate classification of each specimen. Sequence length indicates the number of base pairs that composes each sequence, and sequencing technology indicates whether COI was sequenced with Sanger or NGS. Country and location columns include information on the geographic origin of each specimen, which is essential information for understanding species’ distributions and habitat preferences.

**Table 3.** Summary of the COI data set in the Barcode of Life Data (BOLD) System. Specimens marked with * indicates new record in BOLD.

**BOLD ID**	**Museum ID**	**Identification**	**BIN**	**Sequence length (bp)**	**Sequencing technology**	**Country**	**Location**
PRIS001-24	OMT6	*Sphyrna lewini*	BOLD:AAA2402	652	Sanger	Oman	Mutrah fish market
PRIS002-24	OMT7	*Sphyrna lewini*	BOLD:AAA2402	652	Sanger	Oman	Mutrah fish market
PRIS003-24	ICOS01375	*Erythrocles schlegelii*	BOLD:ADL5501	652	Sanger	Oman	Ra’s al-Hadd
PRIS004-24	ICOS01390	*Epinephelus chlorostigma*	BOLD:AAC5363	652	Sanger	Oman	Raysut
PRIS005-24	ICOS01431	*Rhinobatos punctifer*	BOLD:AAE7441	652	Sanger	Oman	Sour fish market
PRIS006-24	ICOS01432	*Caranx sexfasciatus*	BOLD:AAB0584	652	Sanger	Oman	Sour fish market
PRIS007-24	ICOS02011	*Velifer hypselopterus*	BOLD:AAF1444	1551	NGS	Oman	Salalah fish market
PRIS008-24	ICOS02012	*Ambassis dussumieri*	BOLD:AAJ2348	1539	NGS	Oman	Salalah fish market
PRIS009-24	ICOS02014	*Epinephelus fasciatus*	BOLD:AAB1334	1551	NGS	Oman	Mirbat (fish market)
PRIS010-24	ICOS02015	*Scarus fuscopurpureus*	BOLD:AAD0849	1551	NGS	Oman	Mirbat (port)
PRIS011-24	ICOS02020	*Epinephelus malabaricus*	BOLD:AAB8389	1551	NGS	Oman	Salalah fish market
PRIS012-24	ICOS02022	*Dermatolepis striolata*	BOLD:AAG9989	1551	NGS	Oman	Salalah fish market
PRIS013-24	ICOS02023	*Scarus fuscopurpureus*	BOLD:AAD0849	1551	NGS	Oman	Mirbat (port)
PRIS014-24	ICOS02024	*Leptoscarus vaigiensis*	BOLD:AAD0896	1551	NGS	Oman	Mirbat (port)
PRIS015-24	ICOS02027	*Lutjanus rivulatus*	BOLD:AAB7684	662	Sanger	Oman	Mirbat (port)
PRIS016-24	ICOS02029	*Mustelus mosis*	BOLD:ACF1606	1557	NGS	Oman	Al-Fizayah
PRIS017-24	ICOS02030	*Carcharhinus sorrah*	BOLD:AAA6534	1557	NGS	Oman	Al-Fizayah
PRIS018-24	ICOS02031	*Trachurus indicus*	BOLD:AAA8614	1551	NGS	Oman	Al-Fizayah
PRIS019-24	ICOS02032	Scorpaenopsis lactomaculata*	PRIS019-24	1551	NGS	Oman	Al-Fizayah
PRIS020-24	ICOS02033	*Brotula multibarbata*	BOLD:AAF8818	1551	NGS	Oman	Al-Fizayah
PRIS021-24	ICOS02035	*Hemipristis elongata*	BOLD:AAB8159	1557	NGS	Oman	Salalah fish market
PRIS022-24	ICOS02036	*Trachinotus africanus*	BOLD:AAF5907	1551	NGS	Oman	Salalah fish market
PRIS023-24	ICOS02037	*Lethrinus lentjan*	BOLD:ABZ0131	652	Sanger	Oman	Salalah fish market
PRIS024-24	ICOS02038	*Carcharhinus leiodon*	BOLD:AAA5251	1557	NGS	Oman	Salalah fish market
PRIS025-24	ICOS02039	*Negaprion acutidens*	BOLD:AAC6405	1557	NGS	Oman	Salalah fish market
PRIS027-24	ICOS02133	*Cheilinus undulatus*	BOLD:AAF3078	652	Sanger	Saudi Arabia	Jeddah fish market
PRIS028-24	ICOS02134	*Cetoscarus bicolor*	BOLD:AAE9711	652	Sanger	Saudi Arabia	Jeddah fish market
PRIS029-24	ICOS02135	*Epinephelus fuscoguttatus*	BOLD:AAD8872	652	Sanger	Saudi Arabia	Jeddah fish market
PRIS030-24	ICOS02136	*Gnathanodon speciosus*	BOLD:AAB7462	652	Sanger	Saudi Arabia	Jeddah fish market
PRIS031-24	ICOS02137	*Hipposcarus harid*	BOLD:ADJ9102	652	Sanger	Saudi Arabia	Jeddah fish market
PRIS032-24	ICOS02142	*Scarus ferrugineus*	BOLD:AAC2544	652	Sanger	Saudi Arabia	Jeddah fish market
PRIS033-24	ICOS02146	*Lutjanus gibbus*	BOLD:AAB3276	652	Sanger	Saudi Arabia	Jeddah fish market
PRIS034-24	ICOS02149	*Bolbometopon muricatum*	BOLD:AAF6698	652	Sanger	Saudi Arabia	Jeddah fish market
PRIS035-24	ICOS02150	Plectropomus marisrubri*	BOLD:AAF8874	652	Sanger	Saudi Arabia	Jeddah fish market
PRIS036-24	ICOS02151	*Monotaxis grandoculis*	BOLD:ABZ0166	652	Sanger	Saudi Arabia	Jeddah fish market
PRIS037-24	ICOS02152	*Lutjanus bohar*	BOLD:AAB4501	652	Sanger	Saudi Arabia	Jeddah fish market
PRIS038-24	ICOS02155	*Trachinotus baillonii*	BOLD:AAC0922	634	Sanger	Oman	Mutrah fish market
PRIS040-24	ICOS02159	*Lutjanus ehrenbergii*	BOLD:AAA9577	652	Sanger	Oman	Mutrah fish market
PRIS041-24	ICOS02163	*Lutjanus fulviflamma*	BOLD:ADF5681	652	Sanger	Oman	Mutrah fish market
PRIS042-24	ICOS02165	*Synodus variegatus*	BOLD:AAB5069	652	Sanger	Oman	Mutrah fish market
PRIS043-24	ICOS02166	*Gerres longirostris*	BOLD:ABZ8782	652	Sanger	Oman	Mutrah fish market
PRIS044-24	ICOS02168	*Selar crumenophthalmus*	BOLD:AAB0870	652	Sanger	Oman	Mutrah fish market
PRIS045-24	ICOS02170	*Decapterus russelli*	BOLD:AAB6796	634	Sanger	Oman	Mutrah fish market
PRIS046-24	ICOS02174	*Cheimerius nufar*	BOLD:AAE2592	652	Sanger	Oman	Salalah fish market
PRIS047-24	ICOS02177	Scarus zufar*	BOLD:ABX8543	652	Sanger	Oman	Raysut
PRIS048-24	ICOS02181	*Lethrinus mahsena*	BOLD:AAB6438	652	Sanger	Oman	Mirbat (port)
PRIS049-24	ICOS02183	*Parascolopsis akatamae*	BOLD:AAC6939	652	Sanger	Oman	Salalah fish market
PRIS050-24	ICOS02184	*Epinephelus undulosus*	BOLD:AAE1762	652	Sanger	Oman	Salalah fish market
PRIS051-24	ICOS02187	*Trachinotus blochii*	BOLD:ACF4014	652	Sanger	Oman	Al-Fizayah
PRIS052-24	ICOS02016	*Ostracion cubicus*	BOLD:AAC2246	1551	NGS	Oman	Mirbat (port)
PRIS053-24	ICOS02026	*Scarus ghobban*	BOLD:ABY4451	1551	NGS	Oman	Mirbat (port)

To evaluate this dataset’s contribution to regional barcode coverage, we compared species representation in BOLD at the time of submission. Three species (*Plectropomus marisrubri*, *Scorpaenopsis lactomaculata*, and *Scarus zufar*) had no prior BOLD records and are currently only represented by sequences generated in this study. Several additional taxa remain sparsely represented (fewer than 10 records), including *Bolbometopon muricatum*, *Cetoscarus bicolor*, *Hipposcarus harid*, *Scarus fuscopurpureus*, *Scarus ghobban*, *Trachurus indicus*, and *Velifer hypselopterus*. For *Dermatolepis striolata*, our study increased the number of available records from one to two. These additions demonstrate the value of the dataset in improving taxonomic and geographic coverage for a historically underrepresented region.

## Additional information

### Artificial Intelligence (AI) use

The authors accept full responsibility for the content of the manuscript, including the disclosure of any use of AI.

No AI tools were used in the preparation of this manuscript.

### Author ORCIDs

N.G. Jiménez Cano https://orcid.org/0000-0001-9973-2452

A. Dettaï https://orcid.org/0000-0002-8496-9737

M. Abu El-Regal https://orcid.org/0000-0001-7595-0420

A. Al-Jabri https://orcid.org/0009-0001-6842-4382

P. Béarez https://orcid.org/0000-0003-0397-2393

### Data availability

The data underpinning the analysis reported in this paper are deposited in the Barcode of Life Data System (BOLD; https://boldsystems.org/). The full dataset is available on BOLD at https://doi.org/10.5883/DS-2024. Metadata associated with this paper can be found in Suppl. material 1.

## Supplementary materials

Supplementary material 1 PRISTINE metadata associated with this paper Data type txt https://binary.pensoft.net/file/1765261This dataset is made available under the Open Database License (http://opendatacommons.org/licenses/odbl/1.0/). The Open Database License (ODbL) is a license agreement intended to allow users to freely share, modify, and use this Dataset while maintaining this same freedom for others, provided that the original source and author(s) are credited. Nayeli G. Jiménez Cano
